# LAG3-centered multiplex immune profiling as a prognostic biomarker in hepatocellular carcinoma patients receiving Anti-PD-1 antibodies plus lenvatinib therapy

**DOI:** 10.3389/fonc.2026.1878762

**Published:** 2026-07-01

**Authors:** Juan Zhang, Mingzhen Zhou, Ziyue Jin, Wenyan Cao, Xuena Zhang, Fanlai Meng, Haiyan Wei, Jie Shen

**Affiliations:** The Comprehensive Cancer Center, Department of Oncology, Nanjing Drum Tower Hospital & Group’s Suqian Hospital, Affiliated Hospital of Medical School, Nanjing University, Nanjing, China

**Keywords:** CD8, HCC, LAG-3, lenvatinib plus anti-PD-1 antibodies therapy, PD-1, prognostic biomarkers

## Abstract

**Purpose:**

This study aims to investigate the characteristics and functional status of the lymphocyte-activation gene-3 (LAG-3), cluster of differentiation 8 (CD8), programmed cell death protein-1 (PD-1) immune microenvironment in tumor tissues of hepatocellular carcinoma (HCC) patients and evaluate their value as prognostic biomarkers for the efficacy of combination of anti-PD-1 antibodies with Lenvatinib.

**Patients and methods:**

This retrospective study collected clinical data from 77 patients with hepatocellular carcinoma who received Lenvatinib plus anti-PD-1 antibodies at the Department of Oncology, Nanjing Drum Tower Hospital between January 2018 and June 2023. Following combination therapy with Lenvatinib and anti-PD-1 antibodies, 1 patient achieved complete response (CR), 23 achieved partial response (PR), 39 achieved stable disease (SD), and 14 achieved progressive disease (PD).

**Results:**

Age stratification (<60 years vs ≥60 years), cirrhosis status, and *Eastern Cooperative Oncology Group* (ECOG) performance status (0 vs 1) were statistically associated with treatment response in HCC patients. Multivariate Cox regression analysis revealed that the high pan-immune-inflammation value (PIV) expression group [*Hazard ratio* (HR)=9.239, 95% confidence interval (CI): 1.731-49.310, P = 0.009], the low LAG-3^+^ cell counts group (HR = 0.190, 95% CI: 0.042-0.857, P = 0.031), the low CD8^+^LAG-3^+^ cell density group (HR = 0.198, 95% CI: 0.041-0.962, P = 0.045), the low area proportion of CD8^+^LAG-3^+^ cell group (HR = 5.203, 95% CI: 1.093-24.770, P = 0.038) were independent prognostic risk factors for overall survival (OS) in patients receiving Lenvatinib plus anti-PD-1 antibodies. Independent prognostic risk factors for *progression-free survival* (PFS) in treated patients included the low albumin (ALB) level group (HR = 0.196, 95% CI: 0.042-0.923, P = 0.039) and the high neutrophil-to-lymphocyte ratio (NLR) group (HR = 2.894, 95% CI: 1.076-7.785, P = 0.035).

**Conclusion:**

Our study proposes that LAG-3 serves as a potential prognostic factor for identifying survival benefits from Lenvatinib plus anti-PD-1 antibodies in hepatocellular carcinoma patients.

## 1 Introduction

Hepatocellular carcinoma (HCC) is the sixth most commonly diagnosed malignancy and the fourth leading cause of cancer-related mortality worldwide (1). As the predominant subtype of primary liver cancer, HCC typically develops in the context of chronic liver disease. Well-established etiological risk factors include chronic hepatitis B virus (HBV) or hepatitis C virus (HCV) infection, alcohol-associated liver disease, and non-alcoholic steatohepatitis (NASH) (2). The clinical onset of HCC is usually insidious; accordingly, the majority of patients are diagnosed at an advanced stage (3), Such late diagnosis precludes curative surgical intervention, leaves patients with limited therapeutic options, and ultimately contributes to the poor overall prognosis of HCC patients (4). For advanced HCC, systemic anticancer therapy serves as the cornerstone of standard clinical management. In accordance with the Chinese Society of Clinical Oncology (CSCO) guidelines, the combination of targeted therapy and immunotherapy, represented by the lenvatinib plus anti-PD-1 antibody regimen, is recommended for the treatment of advanced hepatocellular carcinoma.

Consistent with international consensus guidelines, first-line systemic therapy for patients with unresectable advanced HCC includes the combination of atezolizumab and bevacizumab, as reported by Vogel et al. in 2018 (5). The phase III IMbrave150 trial demonstrated that patients treated with the atezolizumab plus bevacizumab regimen achieved a median overall survival (mOS) of 19.2 months, a median progression-free survival (mPFS) of 6.9 months, and an elevated objective response rate (ORR) of 30.0% (6). The randomized, open-label, phase 2–3 ORIENT-32 trial verified that sintilimab combined with a bevacizumab biosimilar yielded significant survival benefits in patients with unresectable HCC compared with sorafenib monotherapy, with an ORR of 20.5% in the combination group (7). The final analysis of the phase 3 CARES-310 trial further confirmed that the camrelizumab plus apatinib regimen achieved a mOS of 23.8 months, a mPFS of 5.6 months, and an ORR of 26.8% as first-line treatment for unresectable hepatocellular carcinoma (8). Collectively, current clinical evidence indicates that combination regimens integrating immunotherapy with targeted therapy exhibit superior efficacy, leading to pronounced improvements in mPFS and mOS among patients with advanced HCC. Nevertheless, the ORR of all aforementioned targeted immunotherapy combinations remains below 30%, highlighting an urgent need to identify reliable prognostic biomarkers for precise patient stratification and optimal selection of individuals most likely to benefit from such combination therapies.

A growing body of research has focused on identifying prognostic biomarkers for therapeutic efficacy in HCC patients, aiming to facilitate precise patient stratification and screen for populations most responsive to treatment. A review published in *Nature Reviews Clinical Oncology* has systematically summarized prognostic and pharmacodynamic biomarkers for single and combination immunotherapy in HCC. The identified biomarkers cover programmed death-ligand 1 (PD-L1), cytotoxic T-lymphocyte-associated protein 4 (CTLA-4), microsatellite instability-high (MSI-H), inflammation-related indicators (e.g., neutrophil-to-lymphocyte ratio [NLR] and platelet-to-lymphocyte ratio [PLR]), circulating tumor DNA (ctDNA) (2), alpha-fetoprotein (AFP) (9), circulating tumor cells (CTCs) (10), and tumor mutational burden (TMB) (11).

Among all investigated biomarkers, inhibitory immune checkpoint receptors have become a major research hotspot due to their robust prognostic potential. Classic biomarkers in this category include PD-1 and CTLA-4, which act as critical negative regulators of T cell-mediated immune responses against normal self-tissues and tumor cells. In addition to PD-1 and CTLA-4, a variety of stimulatory and inhibitory co-receptors participate in the sophisticated modulation of T cell activation. These molecules have been recognized as promising therapeutic targets, driving intensive research and competitive development of novel targeted immunotherapeutic agents. As one of the most promising next-generation immune targets, lymphocyte-activation gene 3 (LAG-3) ranks second only to PD-1 in clinical development priority, with numerous ongoing clinical trials evaluating the efficacy of LAG-3-targeted therapies (12). LAG-3 is an inhibitory immune receptor expressed on multiple immune cells, including activated T cells, regulatory T cells (Tregs), natural killer (NK) cells, plasmacytoid dendritic cells, and a subset of B cells. Its core functional mechanism is binding to the T cell receptor (TCR) complex, thereby transmitting negative signals to suppress T cell proliferation and cytokine secretion (13). By modulating the function of effector T cells, Tregs, and potentially type 1 regulatory T (Tr1) cells, LAG-3 plays a pivotal role in regulating pathological T cell responses, making it a high-value therapeutic target (14). Furthermore, LAG-3 binds to major histocompatibility complex (MHC) class II molecules loaded with stable peptides, which further impairs T cell function and suppresses both autoimmune and anti-tumor immune responses (15).

LAG-3 represents a highly promising immunotherapeutic target, with over 20 investigational agents targeting the LAG-3 pathway currently undergoing clinical evaluation. Upon TCR activation, LAG-3 traffics to the immune synapse and binds to the TCR-CD3 complex on both CD4+ and CD8+ T cells. This molecular interaction restricts proximal TCR signaling and consequently inhibits downstream T cell activation, thereby modulating anti-tumor immune reactivity (16, 17).

Of note, lenvatinib exerts dual anti-tumor effects by not only suppressing tumor angiogenesis but also remodeling the tumor immune microenvironment. It reduces the abundance of immunosuppressive cells, including Tregs and myeloid-derived suppressor cells (MDSCs), thereby establishing an immune-permissive tumor microenvironment that augments the efficacy of subsequent immune checkpoint blockade (29). Mechanistically, PD-1 and LAG-3 synergistically modulate the formation and functional status of exhausted T (Tex) cells: PD-1 primarily inhibits T cell proliferation, whereas LAG-3 specifically restrains the effector functions of CD8^+^ T cells (18). Notably, LAG-3 signaling sustains the exhausted phenotype of CD8^+^ T cells, and its expression level accurately reflects the functional competence of the intratumoral T cell pool (30). Accordingly, we hypothesize that baseline LAG-3 expression on CD8^+^ T cells can reliably indicate the pre-existing T cell exhaustion status within the tumor microenvironment. Under the dual regulatory effects of lenvatinib-induced immune remodeling and PD-1 blockade, elevated LAG-3 expression may identify patients whose intratumoral T cells retain sufficient functional plasticity for immune reinvigoration following combination therapy. This feature renders LAG-3 a specific prognostic biomarker for lenvatinib plus anti-PD-1 combination therapy, rather than a universal predictive indicator for single-agent anti-PD-1 immunotherapy.

Despite the rapid clinical progress of combination immunotherapy regimens, the identification of robust and reliable biomarkers for predicting treatment responses remains a major unmet clinical need for HCC management. Given the well-documented prognostic value of LAG-3 in multiple tumor types, we hypothesized that LAG-3 expression could serve as a promising prognostic biomarker for clinical outcomes in patients with unresectable HCC receiving lenvatinib combined with anti-PD-1 antibody therapy.

Additionally, high intratumoral CD8^+^ T cell infiltration has been validated as an independent prognostic biomarker for favorable clinical outcomes in HCC patients, and its predictive efficacy can be significantly improved when combined with other immune-related biomarkers (2). Currently, effective prognostic biomarkers for targeted-immunotherapy combination regimens in HCC remain insufficiently explored (19), while the tumor immune microenvironment (TIME) has emerged as a critical frontier for prognostic biomarker research in HCC (20). Based on this research background, the present study retrospectively analyzed the OS and PFS data of unresectable HCC patients treated with lenvatinib plus anti-PD-1 antibodies in our center. Multiplex immunofluorescence (mIF) was utilized to quantitatively analyze the expression patterns of key immune checkpoint molecules (LAG-3, PD-1) and the canonical effector T cell marker CD8. Furthermore, we comprehensively evaluated multiple immune parameters, including immune cell proportion, spatial distribution density, and tissue area ratio, to systematically elucidate their potential values in predicting therapeutic responses to lenvatinib plus anti-PD-1 combination therapy.

## 2 Patients and methods

### 2.1 Patients

This retrospective study enrolled patients diagnosed with advanced HCC who received Lenvatinib plus anti-PD-1 antibodies at the Cancer Center of Nanjing Drum Tower Hospital, affiliated with Nanjing University, between January 2018 and June 2023.

The patient inclusion criteria were as follows: (1) histopathologically confirmed intermediate-to-advanced HCC or postoperative recurrent HCC; (2) receipt of first-line combination therapy with lenvatinib plus anti-PD-1 antibodies; (3) age ranging from 18 to 80 years; (4) Eastern Cooperative Oncology Group (ECOG) performance status of 0-1; and (5) presence of at least one measurable lesion evaluated in accordance with the Response Evaluation Criteria in Solid Tumors (RECIST) version 2.1.1 before and after treatment.

Patients were excluded from the study if they met any of the following exclusion criteria: (1) loss of follow-up during the study period; (2) irregular administration of anticancer treatment; (3) absence of valid therapeutic efficacy evaluation data; (4) incomplete baseline clinical or laboratory information; and (5) poor treatment compliance that might substantially interfere with the assessment of therapeutic outcomes.

### 2.2 Clinical data

Baseline demographic, clinical, and pathological data of all enrolled patients were systematically collected. Collected baseline characteristics included age, gender, ECOG performance status, and Child-Pugh classification. Additional clinical variables comprised hepatitis infection status (hepatitis B or hepatitis C infection), liver cirrhosis, smoking history, alcohol consumption history, a history of other malignant tumors, and tumor-specific pathological features including microvascular invasion and macrovascular tumor thrombus. Tumor treatment response was assessed based on the ORR in accordance with the RECIST 1.1 criteria.

All peripheral blood laboratory parameters were acquired from blood samples collected within two weeks prior to treatment initiation. The detected hematological and biochemical indicators included neutrophil count, lymphocyte count, monocyte count, white blood cell (WBC) count, ALB, AFP, and C-reactive protein (CRP). Systemic inflammatory and nutritional prognostic indices were further calculated based on raw laboratory data, including the NLR, PLR, lymphocyte-to-monocyte ratio (LMR), CRP-to-albumin ratio (CAR), lactate dehydrogenase-to-C-reactive protein ratio (LCR), systemic immune-inflammation index (SII), pan-immune-inflammation value (PIV), and prognostic nutritional index (PNI).

### 2.3 Immunofluorescence staining

For all enrolled patients, multiplex immunofluorescence (mIF) staining targeting CD8, PD-1, and LAG-3 was performed on formalin-fixed, paraffin-embedded (FFPE) tumor tissue sections using the PanoVIEW VS200 platform in accordance with a standardized experimental protocol. Whole-slide digital scanning was conducted for all stained sections, followed by quantitative and spatial image analysis to comprehensively characterize the profiles of tumor-infiltrating immune cells. Strict routine quality control was implemented for each staining batch, with parallel positive and negative controls included for validation; only batches with consistent and expected control outcomes were adopted for subsequent analysis. All experimental staining conditions, including antigen retrieval, antibody dilution, incubation duration and temperature, and washing procedures, were strictly standardized and maintained consistent across all experimental runs. Furthermore, representative tissue sections were independently examined by two experienced pathologists to verify staining quality and consistency. Notably, formal quantitative evaluation of inter-run reproducibility, such as the intraclass correlation coefficient (ICC) analysis, was not performed because this assessment was not predefined in the original study protocol; this methodological limitation will be acknowledged and discussed in the Discussion section.

#### 2.3.1 Tissue processing and antigen retrieval

FFPE tumor tissue sections from enrolled patients were first baked at 65 °C for 1 h to enhance tissue adherence. The sections were then deparaffinized in xylene with three successive washes (10 min each), followed by rehydration using a graded ethanol series (100%, 95%, and 70% ethanol for 5 min, 5 min, and 2 min, respectively). After three 5-min rinses with deionized water, the sections were post-fixed in 10% neutral buffered formalin (NBF) for 20 min to preserve tissue antigenicity, and then thoroughly rinsed with deionized water.

#### 2.3.2 Antigen retrieval

Samples were placed in Tris-EDTA buffer and microwaved following a standardized protocol: 100% power for 1 min, followed by 20% power for 15 min. The slides were then cooled naturally to room temperature. After three 5-min washes with deionized water, the sections were immersed in 1× TBST for 2 min.

#### 2.3.3 Blocking

The samples were placed in Tris-EDTA buffer and microwaved per a standardized protocol: 100% power for 1 min, followed by 20% power for 15 min. The slides were then cooled naturally to room temperature. After three 5-min washes with deionized water, the sections were immersed in 1× TBST for 2 min.

#### 2.3.4 Primary antibody incubation

Sections were incubated overnight at 4 °C in a humidified chamber with validated primary antibodies against human CD8, LAG-3 and PD-1. The antibodies used were as follows: anti-CD8 (Cell Signaling Technology, Cat# CST70306), anti-PD-1 (Cell Signaling Technology, Cat# CST86163), and anti-LAG-3 (Cell Signaling Technology, Cat# CST15372). After incubation, unbound antibodies were removed by washing the sections three times for 5 min each in 1× TBST with gentle agitation.

#### 2.3.5 Secondary antibody incubation

Sections were incubated with species-matched fluorescent dye-conjugated secondary antibodies for 1 h at room temperature, protected from light. After incubation, unbound antibodies were eliminated by washing the sections three times for 5 min each in 1× TBST with gentle agitation.

#### 2.3.6 Nuclear staining

Staining was performed using target-specific detection kits for CD8, PD-1, and LAG-3. Each fluorescent dye was incubated for 10–15 min. Between each staining round, the sections were washed three times for 5 min with 1× TBST. Upon completion of the final target detection, cell nuclei were counterstained with 4′,6-diamidino-2-phenylindole (DAPI) via incubation in the dark at room temperature for 10 min. Excess DAPI was removed by three successive 5-min washes with 1× TBST.

#### 2.3.7 Encapsulation and imaging

Anti-fluorescence quenching mounting medium was applied, and the slides were stored in the dark prior to imaging. Whole-slide digital images were acquired using a high-throughput panoramic slide scanner (PanoVIEW VS200). For each fluorescent marker, images were captured with matched dedicated filter sets, including CD8 (red), LAG-3 (yellow), and PD-1 (blue). A representative DAPI channel image was also captured for nuclear localization and identification.

#### 2.3.8 Image and quantitative analysis

Region of interest (ROI) definition for immune cell quantification: Whole-slide digital images of multiplex immunofluorescence-stained sections were acquired using a panoramic pathology workstation (Pano ATLAS). For each slide, a pathologist blinded to clinical outcomes manually annotated the entire viable tumor tissue area as the “Total” region. Based on histomorphological characteristics, the Total region was further subdivided into two mutually exclusive subregions: the tumor region, defined as the core area of tumor cell aggregation excluding stromal components, and the stroma region, defined as the peritumoral and intratumoral stromal compartment composed of immune cells, fibroblasts, and extracellular matrix. After tissue segmentation and cell nucleus identification via DAPI signal, co-expression analysis of fluorescent markers was conducted to phenotype individual cells. A cell was defined as LAG-3^+^/CD8^+^/PD-1^+^ when exhibiting moderate-to-strong staining (intensity score ≥ 2+) in the membranous and/or cytoplasmic compartments. The absolute number of LAG-3^+^ cells within the annotated viable tumor area was automatically enumerated for each sample using integrated digital image analysis software. This value was recorded as the raw count of LAG-3^+^/CD8^+^/PD-1^+^ cells per section and served as the basis for subsequent calculations of cell density (cells/mm^2^) and proportional metrics (e.g., percentages of total immune cells). All quantitative data in this study, including LAG-3^+^ cell counts, CD8^+^LAG-3^+^ cell density, and the area proportion of CD8^+^LAG-3^+^ cells, were derived from the Total region (i.e., the combined tumor and stroma regions), reflecting the overall LAG-3 expression level across the entire tumor tissue. The invasive margin was not analyzed separately, as most specimens were core needle biopsies that failed to consistently capture the complete invasive margin.

### 2.4 Efficacy assessment

Overall tumor responses were evaluated per the RECIST version 1.1, with clinical outcomes categorized as CR, PR, SD, and PD. OS was defined as the time from the initiation of Lenvatinib plus anti-PD-1 antibody combination therapy to death from any cause. Patients who remained alive at the last follow-up were censored at that time point. PFS was defined as the interval from treatment initiation to the first documentation of objective tumor progression per RECIST 1.1, all-cause death, or the last progression-free disease assessment.

### 2.5 Statistical analysis

All statistical analyses were performed using IBM SPSS 26.0, GraphPad Prism 8.0, and X-tile software. X-tile was utilized to determine the optimal cutoff values for target expression levels. Intergroup differences were compared using Pearson’s chi-square test or Fisher’s exact test. For baseline peripheral blood indicators, intergroup comparisons were performed using independent samples t-tests for normally distributed continuous data and Mann-Whitney U tests for non-normally distributed data.

Cells in the HCC immune microenvironment were classified into distinct subsets based on specific marker expression. The positive rates of each cell subset were calculated from multiplex immunofluorescence staining results. X-tile software was further applied to identify the optimal cutoff values of cell subset rates stratified by OS and PFS outcomes, which stratified patients into high- and low-expression groups.

Survival differences between the two groups were analyzed using Kaplan-Meier survival curves and Cox proportional hazards regression models. Univariate Cox regression analysis was conducted to evaluate the effects of various baseline factors on PFS and OS. Variables with severe multicollinearity were excluded. The remaining variables were incorporated into multivariate Cox proportional hazards regression models to screen for independent prognostic predictors of PFS and OS. A two-tailed P-value < 0.05 was considered statistically significant.

## 3 Result

### 3.1 Comparison of clinical characteristics between hepatocellular carcinoma patients with and without disease progression following lenvatinib plus anti-PD-1 antibody treatment

Among the 77 enrolled HCC patients, 1 patient achieved a CR, 23 PR, 39 had SD, and 14 PD. Age stratification (< 60 years vs. ≥ 60 years) was significantly associated with treatment response (χ^2^ = 3.969, *P* = 0.047). Patients aged < 60 years exhibited a significantly higher CR/PR rate (18/45, 40.0%) compared with those aged ≥ 60 years (6/32, 18.8%).

Treatment response also differed significantly according to cirrhosis status (χ^2^ = 8.291, *P* = 0.004). Patients with cirrhosis had a markedly higher CR/PR rate (14/27, 51.9%) than patients without cirrhosis (10/50, 20.0%). Additionally, ECOG performance status (0 vs. 1) was significantly correlated with treatment response (χ^2^ = 3.970, *P* = 0.046). Patients with an ECOG score of 0 demonstrated a higher CR/PR rate (20/52, 38.5%) relative to those with an ECOG score of 1 (4/25, 16.0%). No other baseline clinical characteristics were significantly correlated with treatment response (all *P* > 0.05) (**Table 1**).

**Table 1:** Patient baseline characteristics.

Baseline Characteristics	Total (n=77)	CR/PR (n=24)	SD/PD (n=53)	X^2^	P value
Gender, n(%)					
Male	74 (96.1%)	23(31.1%)	51(68.9%)	/	1.000
Female	3 (3.9%)	1(33.3%)	2(66.7)		
Age, n(%)					
≥60	32 (41.6%)	6(18.8%)	26(81.2%)	3.936	**0.047**
<60	45 (58.4%)	18(40.0%)	27(60.0%)		
Drink,n(%)					
No	59 (76.6%)	17(28.8%)	42(71.2%)	0.653	0.419
Yes	18 (23.4%)	7(38.9%)	11(61.1%)		
Smoke,n(%)					
No	54 (70.1%)	15(27.8%)	39(72.2%)	0.091	0.763
Yes	23 (29.9%)	9(39.1%)	14(60.9%)		
Liver Cirrhosis, n(%)					
No	50 (64.9%)	10(20.0%)	40(80.0%)	8.291	**0.004**
Yes	27 (35.1%)	14(51.9%)	13(48.1%)		
History of the tumor, n(%)					
No	75 (97.4%)	24(32.0%)	51(68.0%)	/	1.000
Yes	2 (2.6%)	0(0.0%)	2(100.0%)		
HBV/HCV infection, n(%)					
Yes	64 (83.1%)	23(35.9%)	41(64.1%)	/	0.053
No	13 (16.9%)	1(7.7%)	12(92.3%)		
AFP(ng/L), n(%)					
≥400	31(40.3%)	8(25.8%)	23(74.2%)	0.696	0.404
<400	46(59.7%)	16(34.8%)	30(65.2%)		
Child-Pugh, n(%)					
A	74(96.1%)	23(31.1%)	51(68.9%)	/	1.000
B	3(3.9%)	1(33.3%)	2(66.7%)		
ECOG, n(%)					
0	52 (67.5%)	20(38.5%)	32(61.5%)	3.970	**0.046**
1	25 (32.5%)	4(16.0%)	21(84.0%)		
Microvascular invasion, n(%)					
Non-existence	57 (74.0%)	18(31.6%)	39(68.4%)	0.017	0.896
Existence	20 (26.0%)	6(30.0%)	14(70.0%)		
Macrovascular invasion, n(%)					
Non-existence	61 (79.2%)	18(29.5%)	43(70.5%)	/	0.556
Existence	16 (20.8%)	6(37.5%)	10(62.5%)		
CNLC stage, n(%)					
II	13 (16.9%)	5(38.5%)	8(61.5%)	/	0.529
III	64 (83.1%)	19(29.7%)	45(70.3%)		

Fisher’s exact test. Bold values indicate statistical significance (P < 0.05).

### 3.2 Association between peripheral blood parameters and response to lenvatinib plus anti-PD-1 antibody therapy

**Figure 1:**
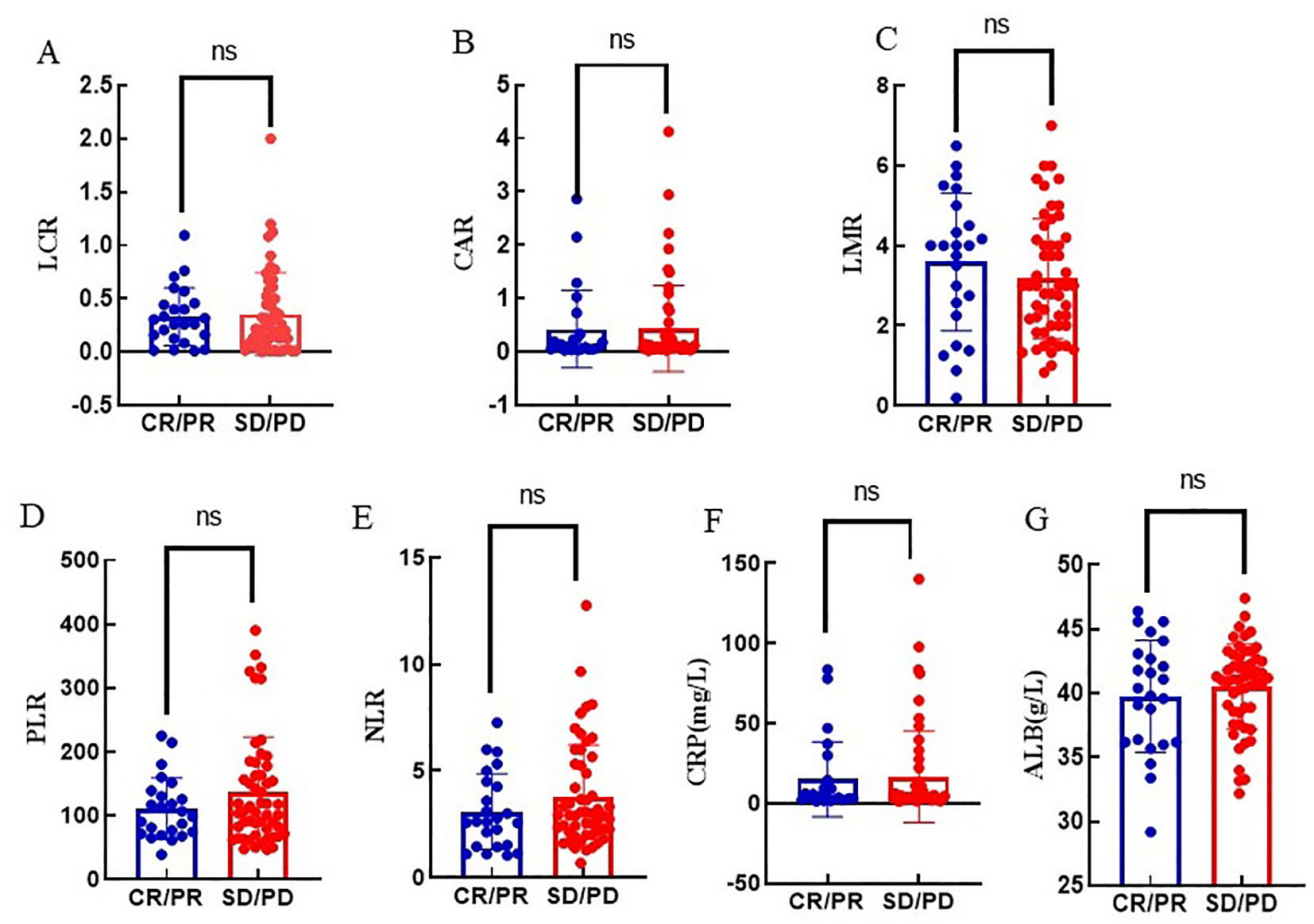
Comparison of baseline peripheral blood parameters between CR/PR and SD/PD patients; **(A)** LCR, **(B)** CAR, **(C)** LMR, **(D)** PLR, **(E)** NLR, **(F)** CRP, **(G)** ALB. Note: Mann-Whitney U test was used for LCR, CAR, PLR, NLR, and CRP; independent samples t-test was used for LMR and ALB.

Among the 77 HCC patients receiving the antitumor regimen, 1 achieved CR, 23 achieved PR, 39 achieved SD, and 14 achieved PD. Comparison of baseline peripheral blood inflammatory markers between the CR/PR and SD/PD groups revealed no significant intergroup differences (all *P* > 0.05; **Figure 1**).

### 3.3 Detection of immune-related molecules in HCC tissues via immunofluorescence staining

**Figure 2:**
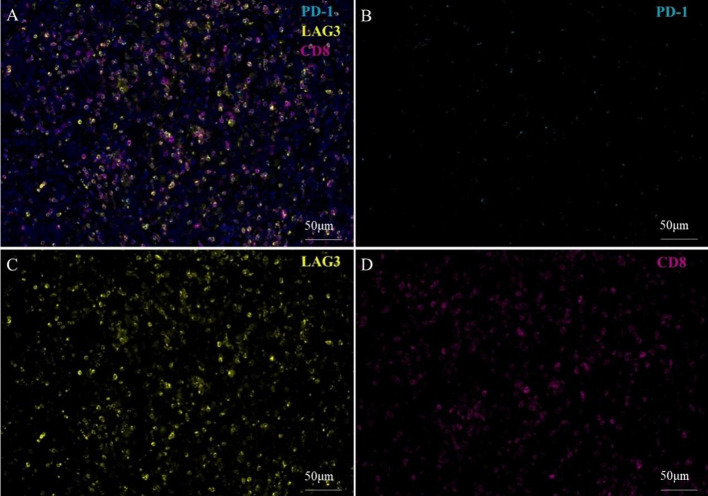
Multiplex immunofluorescence images of CD8, PD-1 and LAG-3 in tumor tissues. **(A)** Merged image showing the colocalization of CD8, PD-1 and LAG-3. **(B)** Single-channel image of PD-1 (blue fluorescence). **(C)** Single-channel image of LAG-3 (yellow fluorescence). **(D)** Single-channel image of CD8 (red fluorescence).

Immunofluorescence staining was performed to detect the expression of immune-related molecules in HCC tissues and characterize the cellular subset distribution within the tumor immune microenvironment. The positive rates of targeted cell subsets were calculated, and their correlations with OS and PFS were further analyzed. The staining assay was mainly used to evaluate the expression of CD8, PD-1, and LAG-3 (**Figure 2**).

#### 3.3.1 Quantitative profiling of immune cell subsets in the tumor microenvironment

To characterize the pretreatment immune landscape of HCC tumors, we performed a quantitative analysis of CD8^+^, PD-1^+^, LAG-3^+^ single-positive cells and their co-expressed subsets via multiplex immunofluorescence (mIF) staining on FFPE tumor sections. All quantitative results are summarized in **Table 2**.

**Table 2:** Mann-Whitney U test results for all eight parameters.

Parameter	Mann-Whitney U	Wilcoxon W	Z	P-value (two-tailed)
LAG-3^+^ cell density	897.000	1197.000	2.870	**0.004**
CD8^+^LAG-3^+^ cell density	802.500	1102.500	1.831	0.067
LAG-3^+^PD-1^+^ cell density	771.000	1071.000	1.488	0.137
CD8^+^LAG-3^+^PD-1^+^ cell density	679.000	979.000	0.476	0.634
Area proportion of LAG-3^+^ cell	884.000	1184.000	2.727	**0.006**
Area proportion of CD8^+^LAG-3^+^ cell	767.500	1067.500	1.446	0.148
Area proportion of LAG-3^+^PD-1^+^ cell	759.500	1059.500	1.361	0.173
Area proportion of CD8^+^LAG-3^+^PD-1^+^ cell	675.000	975.000	0.432	0.666

Bold p-values indicate statistical significance at α = 0.05. Total N = 77 (Group 1: n = 24, Group 0: n = 53). Bold values indicate statistical significance (P < 0.05).

In the total tumor region, the median density of LAG-3^+^ cells was 5.14 cells/mm^2^ (IQR: 1.62-18.97) in non-responders and 17.15 cells/mm^2^ (IQR: 4.83-65.95) in treatment responders. For CD8^+^LAG-3^+^ double-positive cells, the median density was 1.49 cells/mm^2^ (IQR: 0.38-4.28) in non-responders and 3.10 cells/mm^2^ (IQR: 1.18-7.58) in responders. The median density of LAG-3^+^PD-1^+^ double-positive cells was 2.56 cells/mm^2^ (range: 0-161.60) in non-responders and 1.25 cells/mm^2^ (IQR: 0.23-3.66) in responders. Triple-positive (CD8^+^LAG-3^+^PD-1^+^) cells were sparsely distributed in tumor tissues, with a median density of 0.119 cells/mm^2^ (IQR: 0-0.119) in non-responders and 1.32 cells/mm^2^ (IQR: 0-1.26) in responders.

Group comparison between responders (CR/PR, n = 24) and non-responders (SD/PD, n = 53) revealed that responders exhibited significantly higher LAG-3^+^ cell density (P = 0.004, Mann-Whitney U test). In contrast, no significant intergroup differences were observed in the densities of CD8^+^LAG-3^+^ cells (P = 0.067), LAG-3^+^PD-1^+^ cells (P = 0.137), or CD8^+^LAG-3^+^PD-1^+^ triple-positive cells (P = 0.634). Consistently, the area proportion of LAG-3^+^ cells relative to the total tumor area was markedly elevated in responders (median: 0.00239% vs. 0.00063%, P = 0.006). No significant differences were detected in the area proportions of CD8^+^LAG-3^+^ cells (P = 0.148), LAG-3^+^PD-1^+^ cells (P = 0.173), or CD8^+^LAG-3^+^PD-1^+^ cells (P = 0.666). Representative images showing the spatial distribution of these immune cell subsets are presented in **Figure 2**, and the full quantitative dataset is available in **Table 2**.

Furthermore, Mann-Whitney U test was applied to compare eight immune cell-related parameters between the two groups (responders, n = 24; non-responders, n = 53). Statistical analyses confirmed significant differences in LAG-3^+^ cell density (U = 897.000, Z = 2.870, P = 0.004) and LAG-3^+^ cell area proportion (U = 884.000, Z = 2.727, P = 0.006). The remaining six immune parameters showed no significant intergroup differences (all P > 0.05). Detailed statistical results are summarized in **Table 1**.

### 3.4 Prognostic factors affecting PFS and OS in patients receiving Lenvatinib plus anti-PD-1 antibodies

Cellular subpopulations in the immunological microenvironment of hepatocellular carcinoma were classified according to specific markers, and the positivity rate of each subpopulation was calculated based on immunofluorescence staining results. Using X-tile software, optimal cutoff values for the proportions of each cell subset were determined using PFS, OS, baseline clinical characteristics, and peripheral blood indicators. All patients were subsequently stratified into high- and low-level groups. The optimal cutoff values derived from OS and PFS are summarized in **Supplementary Table 1**. Survival relationships between the two groups were compared using Kaplan-Meier curves, with log-rank tests applied for comparing the two survival curves. The cutoffs were determined in an exploratory manner within this discovery cohort and require validation in independent external cohorts.

**Figure 3:**
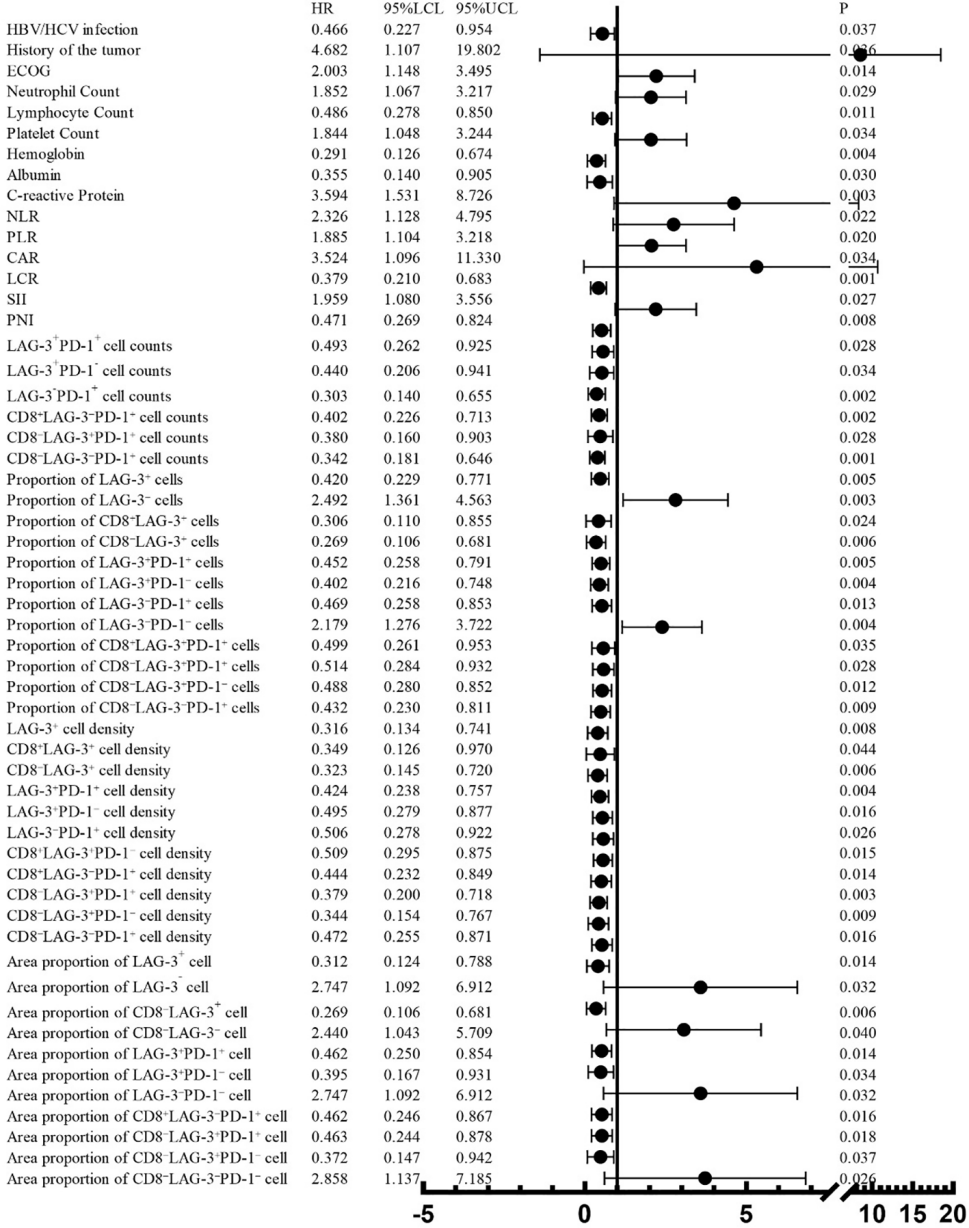
Forest plot of univariate Cox regression analysis for PFS in hepatocellular carcinoma patients receiving targeted plus immunotherapy.

Based on the results of univariate Cox proportional hazards regression analysis, statistically significant risk factors (*P* < 0.05) affecting PFS in patients treated with lenvatinib plus anti-PD-1 antibody combination therapy were identified. These factors were visualized via a forest plot (**Figure 3**). The forest plot adopts HR = 1 as the reference line and visually presents the hazard ratio (HR), 95% confidence interval (CI), and corresponding *P*-value for each candidate prognostic factor. HR values and 95% CIs lying entirely to the right of the reference line (HR > 1) indicate unfavorable prognostic risk factors. Conversely, HR values and 95% CIs lying entirely to the left of the reference line (HR < 1) indicate favorable protective factors. HR values and 95% CIs spanning the reference line (HR = 1) indicate no statistically significant correlation with patient prognosis. This analysis provides a reliable basis for variable screening in the subsequent multivariate Cox regression analysis.

**Figure 4:**
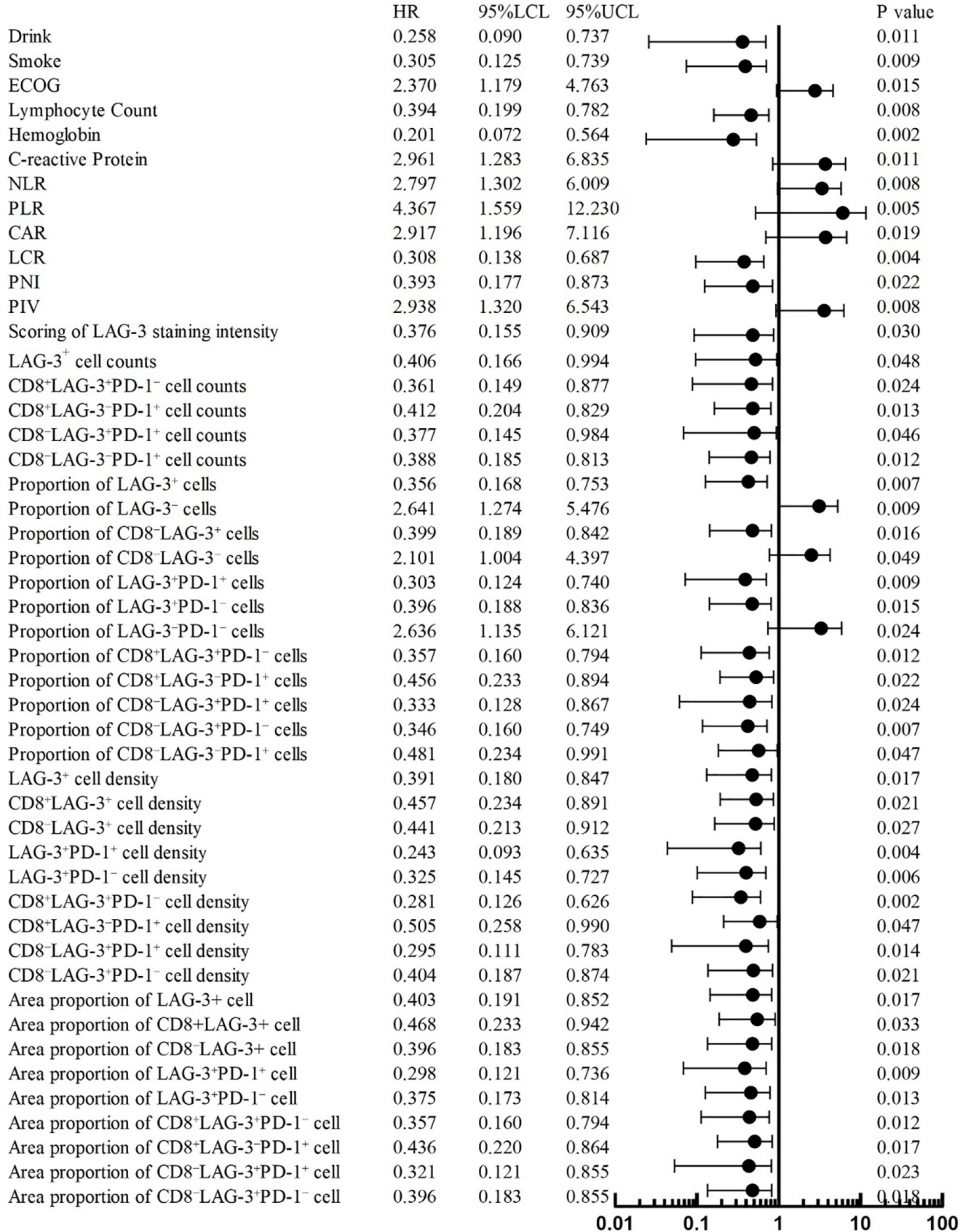
Forest plot of univariate Cox regression analysis for OS in hepatocellular carcinoma patients receiving targeted combined immunotherapy.

Similarly, univariate Cox proportional hazards regression analysis was conducted to screen for factors associated with OS in patients receiving lenvatinib plus anti-PD-1 antibody therapy. Variables with *P* < 0.05 were screened out, and a forest plot (**Figure 4**) was generated to visualize their effect sizes and statistical significance.

Multivariate Cox regression analysis was performed to screen for independent prognostic factors associated with OS and PFS. Prior to modeling, collinearity diagnostics were conducted based on the variance inflation factor (VIF). Variables with VIF > 10 and tolerance < 0.1 were excluded from subsequent modeling to eliminate multicollinearity interference. The remaining variables were included in the final multivariate regression model to determine independent factors affecting OS and PFS.

Multivariable Cox proportional hazards regression analysis was performed incorporating variables that were statistically significant in univariate analysis along with clinically relevant factors. The multivariable Cox regression analysis based on OS showed that PIV (HR = 9.239, 95% CI: 1.731-49.310, P = 0.009) and area proportion of CD8^+^LAG-3^+^ cell (HR = 5.203, 95% CI: 1.093-24.770, P = 0.038) were independent risk factors for patient prognosis, whereas LAG-3^+^ cell counts (HR = 0.190, 95% CI: 0.042-0.857, P = 0.031) and CD8^+^LAG-3^+^ cell density (HR = 0.198, 95% CI: 0.041-0.962, P = 0.045) were independent protective factors. Based on PFS, multivariable Cox regression analysis adjusting for other covariates revealed that ALB (HR = 0.196, 95% CI: 0.042-0.923, P = 0.039) was an independent protective factor, whereas NLR (HR = 2.894, 95% CI: 1.076-7.785, P = 0.035) was an independent risk factor for patient prognosis. It should be noted that the confidence intervals for several hazard ratio estimates were wide, reflecting limited precision due to the modest sample size; accordingly, these findings should be interpreted as exploratory.

**Figure 5:**
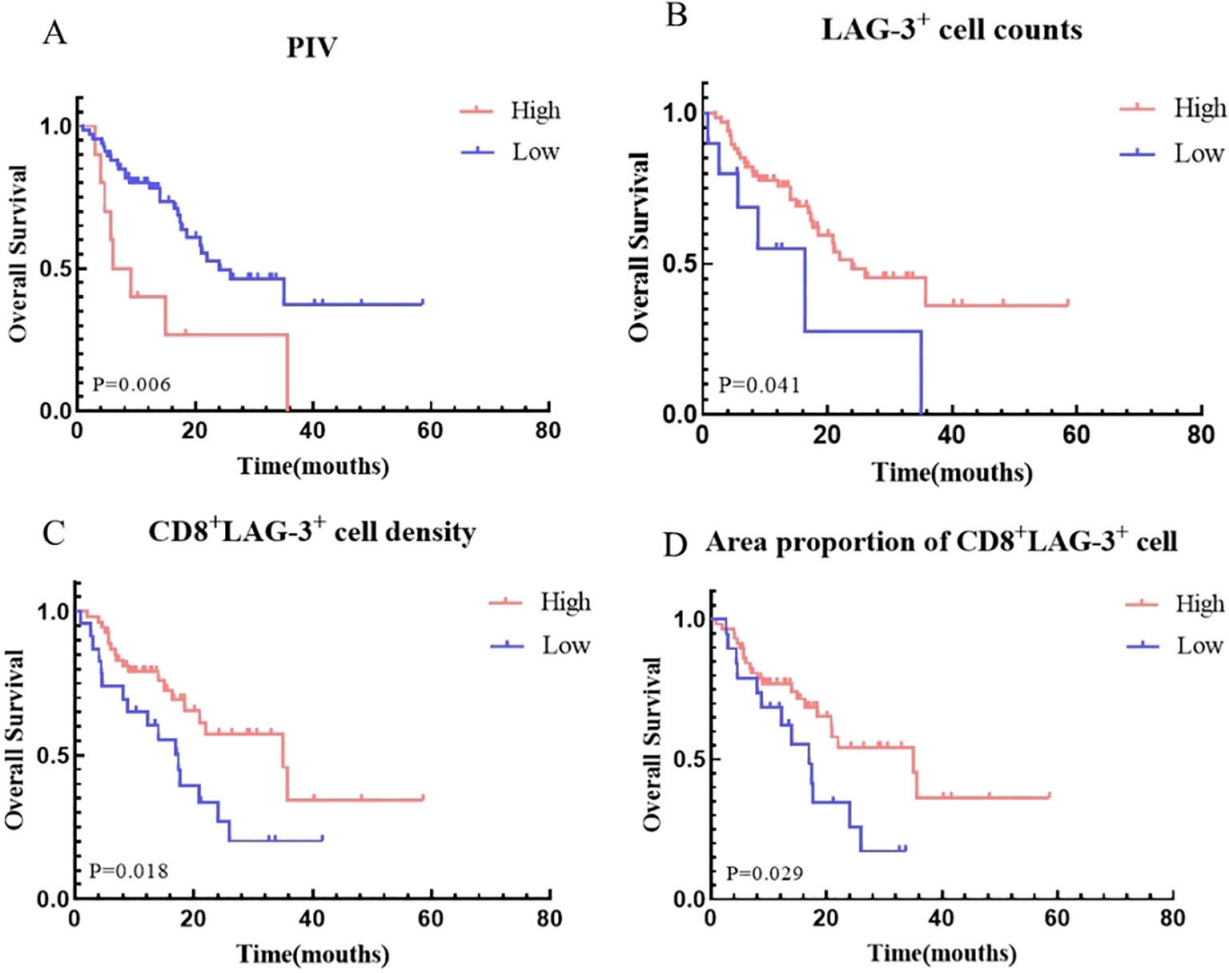
Kaplan-Meier survival curves for different peripheral blood levels and cell subset rates in patients. **(A)** OS curves for PIV; **(B)** OS curves for LAG-3^+^ cell counts; **(C)** OS curves for CD8^+^LAG-3^+^ cell density; **(D)** OS curves for area proportion of CD8^+^LAG-3^+^ cell.

**Figure 6:**
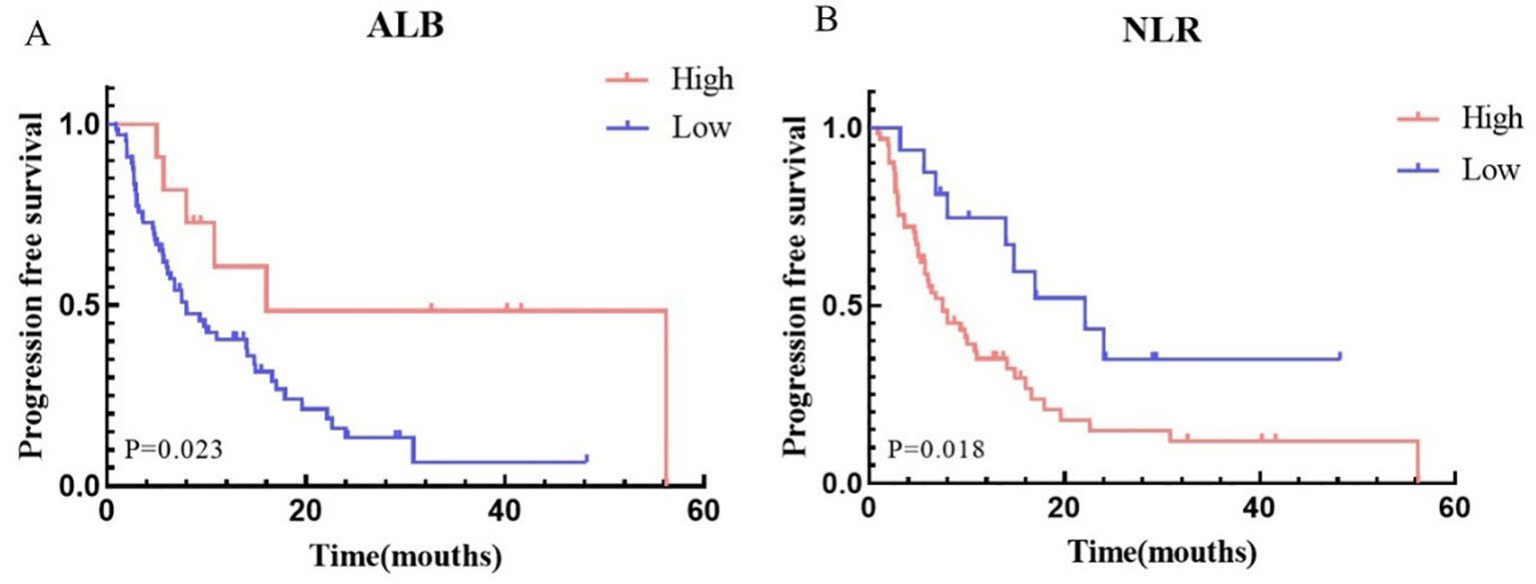
Kaplan-Meier survival curves for different peripheral blood levels in patients. **(A)** PFS curves for ALB; **(B)** PFS curves for NLR.

Kaplan-Meier survival analyses further illustrated the prognostic associations. As shown in **Figure 5A**, mOS was significantly shorter in the high PIV level group (>575.15) [6.1 months (95% CI: 0.957-11.183) vs. 24.0 months (95% CI: 12.420-35.580), *P* = 0.006]. In terms of tumor immune markers, patients in the low LAG-3^+^ cell count group (≤7) exhibited shorter mOS than those in the high LAG-3^+^ group (>7) [16.4 months (95% CI: 5.5-27.3) vs. 24.0 months (95% CI: 13.3-34.7), *P* = 0.041; **Figure 5B**]. Survival analysis based on CD8^+^LAG-3^+^ cell density grouping (**Figure 5C**) revealed that patients in the high CD8^+^LAG-3^+^ cell density group (>0.72) had an mOS of 35.0 months (95% CI: 20.495-49.505), which was significantly longer than 17.4 months (95% CI: 10.571-24.229) in the low-density group (≤0.72). The overall median OS of the entire cohort was 22 months (95% CI: 13.958-30.042). Log-rank tests confirmed significant differences in survival distributions between the two groups (*P* = 0.018), indicating that high CD8^+^LAG-3^+^ cell density serves as a favorable prognostic factor for patient survival. Patients in the group with a low area proportion of CD8^+^LAG-3^+^ cells (≤5.83×10^-5^) exhibited significantly shorter mOS relative to the high group (>5.83×10^-5^) [17.0 months (95% CI: 11.048-22.952) vs. 35.0 months (95% CI: 20.695-49.305), *P* = 0.029; **Figure 5D**]. Collectively, these findings suggest that elevated levels of LAG-3^+^ and CD8^+^LAG-3^+^ cells are associated with favorable survival outcomes in this patient cohort. Notably, the relatively wide confidence intervals of the results warrant cautious interpretation. For PFS, multivariate Cox regression analysis further identified factors associated with progression-free survival. Similarly, multivariate Cox regression analysis for PFS showed that the low ALB group (≤43.7) had shorter mPFS [8.0 months (95% CI: 4.816-11.184) vs. 16.0 months (95% CI: 0.0-40.394), *P* = 0.023], as presented in **Figure 6A**. Survival analysis stratified by NLR (**Figure 6B**) showed that patients in the low-level group (≤1.93) had an mPFS of 22.1 months (95% CI: 10.029-34.171), which was markedly longer than 7.5 months (95% CI: 5.178-9.822) in the high-level group (>1.93). The median PFS of the overall cohort was 9.3 months (95% CI: 5.952-12.648). Log-rank tests identified a significant difference in survival distributions between the two groups (P = 0.018). Of note, the wide confidence intervals for median PFS estimates, particularly in the high ALB group (0.0-40.394 months), reflect limited statistical precision, which may be attributed to the moderate sample size and censored data. Therefore, these findings should be considered exploratory, suggesting that low NLR levels may correlate with favorable PFS in this cohort; however, these results require cautious interpretation.

Pretreatment mIF staining targeting CD8/PD-1/LAG-3 in 77 patients with advanced HCC enabled digital quantification of cell counts, cell density, and area percentage. Low LAG-3^+^ cell counts, low cell density, and low cellular area percentage of CD8^+^LAG-3^+^ cells defined a favorable prognostic subgroup, highlighting the prognostic relevance of spatial immune phenotyping.

## 4 Discussion

Numerous clinical studies have identified a variety of prognostic biomarkers closely correlated with treatment outcomes in HCC. These biomarkers can effectively predict treatment responses and long-term patient survival, thereby playing a pivotal role in optimizing individualized therapeutic regimens. However, due to the high heterogeneity and complex pathogenesis of HCC, accurate prognostic prediction remains a formidable challenge (21). The clinical demand for reliable HCC biomarkers has not been fully addressed, and efficient biomarkers for predicting responses to lenvatinib plus anti-PD-1 antibody therapy in advanced HCC are urgently required to screen patients with unresectable advanced HCC who are most likely to benefit from this combination treatment.

**Figure 7:**
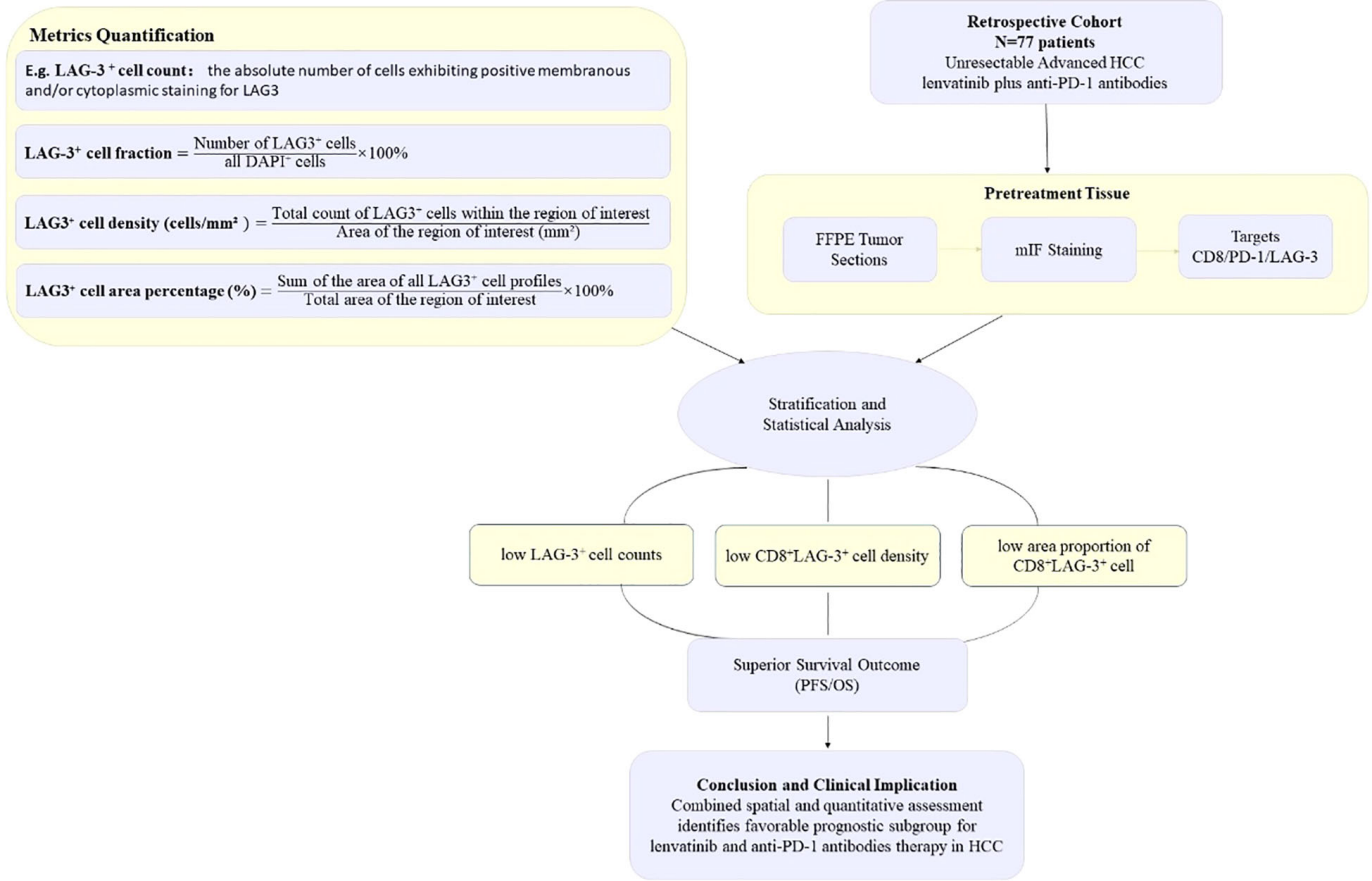
Study design and analytical workflow for spatial profiling of CD8^+^/PD-1^+^/LAG-3^+^ T cells in unresectable advanced HCC.

This study comprehensively analyzed clinical data from 77 patients with HCC who received combined targeted and immunotherapy (**Figure 7**). The results revealed that patients with low LAG-3^+^ cell counts (HR = 0.190, 95% CI: 0.042-0.857, P = 0.031), low CD8^+^LAG-3^+^ cell density (HR = 0.198, 95% CI: 0.041-0.962, P = 0.045), and low area proportion of CD8^+^LAG-3^+^ cells (HR = 5.203, 95% CI: 1.093-24.770, P = 0.038) exhibited significantly shortened OS. These immune-related indicators were further validated as independent risk factors for poor prognosis in HCC patients. Collectively, these findings suggest that LAG-3 and CD8 may serve as promising prognostic biomarkers for the efficacy of lenvatinib plus anti-PD-1 antibody therapy in HCC. Specifically, HCC patients with high LAG-3 expression on tumor-infiltrating CD8^+^ T cells achieve favorable survival outcomes following this combination therapy. The potential core mechanism may be attributed to synergistic therapeutic effects: targeted therapy remodels the tumor microenvironment (22) while immunotherapy abrogates LAG-3-mediated suppression of CD8^+^ T cells. This dual effect reactivates LAG-3^+^CD8^+^ T cells from a dysfunctional but reversible state into potent effector cells with robust antitumor activity (23).

Accumulating clinical and preclinical evidence supports the prognostic value of LAG-3 in tumor immunotherapy. A multicenter phase Ib/II trial enrolling 80 patients with advanced standard therapy-refractory solid tumors demonstrated differential therapeutic efficacy of combined anti-LAG-3 (LBL-007) and anti-PD-1 (toripalimab) treatment stratified by LAG-3 expression. Patients with high tumor LAG-3 expression (defined as IHC score ≥ 2+) achieved a significantly higher ORR of 28.0%, compared with only 7.7% in the low-expression group, indicating that elevated LAG-3 expression is correlated with improved treatment responses and prolonged survival (24). Consistent with these clinical findings, a preclinical study of combination immunotherapy reported that the high LAG-3 expression subgroup exhibited a 40% higher objective response rate and significantly prolonged survival (HR = 0.32) relative to controls, further validating the prognostic significance of LAG-3 upregulation in combination immunotherapy (25). These results are highly consistent with our study conclusion that HCC patients with high LAG-3 expression derive greater clinical benefits from lenvatinib plus anti-PD-1 antibody therapy.

Notably, cell density quantifies the numerical abundance of double-positive immune cells in the TME (26), whereas area percentage reflects the spatial distribution and infiltration degree of these cells (27), representing essential and complementary dimensional differences between the two indicators. Integrating these two metrics captures quantitative and spatial features of CD8^+^LAG-3^+^ cells, enabling precise prognostic stratification and helping identify HCC patients most likely to benefit from combined targeted therapy and immunotherapy.

Of note, our study observed higher treatment response rates in patients with cirrhosis, which appears to contradict previous studies reporting that cirrhosis impairs therapeutic efficacy and reduces survival benefits in HCC patients (28). This discrepancy may arise from the small cirrhosis subgroup (n=27), which limits generalizability, and the predominance of well-compensated liver function (Child-Pugh A, 96.1%). In these patients, preserved hepatic function and drug metabolism may offset the detrimental effects of cirrhosis, suggesting that lenvatinib plus anti-PD-1 offers particular benefit in compensated cirrhosis. Prospective studies are needed to confirm this hypothesis.

Despite demonstrating the prognostic utility of TME-related indicators in advanced HCC treated with lenvatinib plus anti-PD-1, this study has limitations. These include a single-center design with limited sample size; reliance on multiplex immunofluorescence without validation by flow cytometry or single-cell sequencing; lack of formal inter-run reproducibility assessment for staining; and the single-arm design, which precludes determining whether LAG-3 expression is a predictive marker specific to this combination or a general prognostic factor. A comparator arm is needed to establish predictive value.

Based on the limitations of this study, future investigations can be further pursued in the following direction: multi-center and large-sample validation: Implement multi-center, prospective cohort studies to expand the sample size, include formal reproducibility analyses to further validate the reliability of the immune profiling results, and incorporate comparator treatment arms to determine the predictive versus prognostic nature of LAG-3 expression.

Optimal cutoffs for continuous variables were determined by X-Tile using the discovery cohort (n=77). This single-dataset optimization may introduce optimism bias and overfitting. Internal validation was omitted due to limited sample size, as resampling methods produce unstable estimates in small cohorts. Therefore, these findings are exploratory and require external validation in larger, multicenter cohorts before clinical application.

Notably, the hazard ratio for CD8^+^LAG-3^+^ area proportion reversed in the fully adjusted multivariate model compared with Kaplan-Meier and univariate analyses. This reversal likely reflects overadjustment for an immune-related mediator on the causal pathway of the biomarker. When such a mediator is included, the estimated direct effect can become unstable and may even change sign, even if VIF shows no severe multicollinearity. Therefore, the protective association in Kaplan-Meier analysis and mediator-unadjusted models more reliably reflects the biomarker’s true prognostic impact.

Multiplex immunofluorescence staining was conducted by a collaborating laboratory. While routine quality controls (positive/negative controls, standardized protocols and visual inspection) were implemented, formal quantitative assessment of inter-run reproducibility (e.g., ICC) was not performed. This restricts the evaluation of the staining assay’s precision. Future studies should incorporate such reproducibility analyses to further verify the reliability of immune profiling outcomes.

The mechanism linking low baseline LAG-3 on CD8^+^ T cells to favorable outcomes remains unclear. We hypothesize low LAG-3 reflects early, reversible exhaustion, allowing lenvatinib-induced Treg/MDSC reduction to reinvigorate these cells and potentiate PD-1 blockade. High LAG-3 may denote terminal exhaustion. These findings provide prognostic biomarkers that may guide individualized treatment and risk stratification in advanced HCC, pending validation by single-cell and functional assays.

## Data Availability

The original contributions presented in the study are included in the article/**Supplementary Material**. Further inquiries can be directed to the corresponding authors.

## Glossary

LAG-3: Lymphocyte-activation gene-3
HCC: Hepatocellular carcinoma
CD8: Cluster of differentiation 8
CR: Complete response
PR: Partial response
SD: Stable disease
PD: Progressive disease
OS: Overall survival
PFS: *Progression-free survival*
PD-1: Programmed death-1
ECOG: *Eastern Cooperative Oncology Group*
PIV: Pan-immune-inflammation value**;** HR, *Hazard ratio*
CI: Confidence interval
HBV: Hepatitis B virus
HCV: Hepatitis C virus
NASH: Non-alcoholic steatohepatitis
CSCO: Chinese Society of Clinical Oncology
TKIs: Tyrosine kinase inhibitors
VEGF: *Vascular endothelial growth factor*
PD-L1: *Programmed cell death ligand 1*
mPFS: Median progression-free survival
mOS: Median overall survival
CTLA-4: Cytotoxic T-lymphocyte-associated protein 4
Tregs: Regulatory T cells
NK: Natural killer
Tr1: Type 1 regulatory T
MHC: Major histocompatibility complex
Tex: Exhausted CD8^+^ T cells
TCR: T-cell receptor
RECIST: Response Evaluation Criteria in Solid Tumors
FFPE: Formalin-fixed, paraffin-embedded
ORR: Objective response rate
MDSC: Myeloid-derived suppressor cell (MDSCs
ALB: Albumin
AFP: Alpha-fetoprotein
MSI-H: Microsatellite instability-high
ctDNA: circulating tumor DNA
TIME: Tumor immune microenvironment
mIF: multiplex immunofluorescence
WBC: White blood cell
CRP: C-reactive protein
NLR: Neutrophil-to-lymphocyte ratio
PLR: Platelet-to-lymphocyte ratio
LMR: Lymphocyte-to-monocyte ratio
CAR: C-reactive protein-to-albumin ratio
LCR: Lactate dehydrogenase-to-C-reactive protein ratio
PNI: *Prognostic nutritional index*
SII: Systemic immune-inflammation index
NBF: Neutral buffered formalin
DAPI: 4′,6-diamidino-2-phenylindole
VIF: Variance inflation factor
CTCs: Circulating tumor cells
TME: Tumor microenvironment
TMB: Tumor mutational burden
IHC: Immunohistochemistry
